# Monoclonal antibodies targeting cell surface deposited complement fragment C3d potentiate cancer immunotherapy and eliminate antigen loss variants

**DOI:** 10.1186/2051-1426-3-S2-P372

**Published:** 2015-11-04

**Authors:** Martin Skarzynski, Margeret Lindorfer, Vicent Butera, Berengere Vire, Mohammed Farooqui, Christoph Rader, Ronald Taylor, Adrian Wiestner

**Affiliations:** 1 NHLBI, Bethesda, MD, USA; 2 University of Virginia, Charlottesville, VA, USA; 3 Scripps Florida, Jupiter, FL, USA

## 

Treatment-induced loss of targeted cell surface antigens through trogocytosis or internalization reduces efficacy of monoclonal antibody (mAb) therapy of cancer. However, cells that escape therapy mediated by complement-fixing mAbs carry covalently deposited complement activation fragments on their cell surfaces, in particular C3d. We hypothesized that cell-associated C3d constitutes a neoantigen that could be exploited to selectively retarget cells escaping from therapeutic mAbs. We generated an anti-C3d IgG1 human/mouse chimeric mAb specific for human C3d that is not competed by full-length C3 in human serum.

We then set out to provide proof for the concept that complement-targeting mAbs can retarget cancer cells that survive mAb therapy. For this purpose, we used cells from chronic lymphocytic leukemia (CLL) patients that had substantially reduced CD20 levels due to *in vivo* treatment with the anti-CD20 mAb ofatumumab (OFA). The chimeric anti-C3d mAb bound cell surface C3d on these CLL cells *ex vivo* (K_D_ = 6.7nM), and mediated complement-dependent, and antibody-dependent cellular cytotoxicity and phagocytosis *in vitro*. CLL cells opsonized by C3d *in vivo* and reacted with the anti-C3d mAb *in vitro* were further C3d opsonized, resulting in an amplification that enhanced anti-C3d mAb binding capacity and killing of target cells.

*In vivo*, the anti-C3d mAb was effective in reducing tumor growth and extending survival in a mantle cell lymphoma xenograft mouse model. This complement-targeting mAb also depleted human primary CLL cells in the blood and spleens of xenografted NSG mice. Our results identify anti-C3d mAbs as a means to circumvent antigen loss by specifically and potently augmenting the therapeutic efficacy of complement-fixing mAbs.

